# Design of composite measure schemes for comparative severity assessment in animal-based neuroscience research: A case study focussed on rat epilepsy models

**DOI:** 10.1371/journal.pone.0230141

**Published:** 2020-05-15

**Authors:** Roelof Maarten van Dijk, Ines Koska, Andre Bleich, Rene Tolba, Isabel Seiffert, Christina Möller, Valentina Di Liberto, Steven Roger Talbot, Heidrun Potschka

**Affiliations:** 1 Institute of Pharmacology, Toxicology and Pharmacy, Ludwig-Maximilians-University, Munich, Germany; 2 Institute for Laboratory Animal Science and Central Animal Facility, Hannover Medical School, Hannover, Germany; 3 Department of Experimental Biomedicine and Clinical Neurosciences, University of Palermo, Palermo, Italy; University of Modena and Reggio Emilia, ITALY

## Abstract

Comparative severity assessment of animal models and experimental interventions is of utmost relevance for harm-benefit analysis during ethical evaluation, an animal welfare-based model prioritization as well as the validation of refinement measures. Unfortunately, there is a lack of evidence-based approaches to grade an animal’s burden in a sensitive, robust, precise, and objective manner. Particular challenges need to be considered in the context of animal-based neuroscientific research because models of neurological disorders can be characterized by relevant changes in the affective state of an animal. Here, we report about an approach for parameter selection and development of a composite measure scheme designed for precise analysis of the distress of animals in a specific model category. Data sets from the analysis of several behavioral and biochemical parameters in three different epilepsy models were subjected to a principal component analysis to select the most informative parameters. The top-ranking parameters included burrowing, open field locomotion, social interaction, and saccharin preference. These were combined to create a composite measure scheme (CMS). CMS data were subjected to cluster analysis enabling the allocation of severity levels to individual animals. The results provided information for a direct comparison between models indicating a comparable severity of the electrical and chemical post-status epilepticus models, and a lower severity of the kindling model. The new CMS can be directly applied for comparison of other rat models with seizure activity or for assessment of novel refinement approaches in the respective research field. The respective online tool for direct application of the CMS or for creating a new CMS based on other parameters from different models is available at https://github.com/mytalbot/cms. However, the robustness and generalizability needs to be further assessed in future studies. More importantly, our concept of parameter selection can serve as a practice example providing the basis for comparable approaches applicable to the development and validation of CMS for all kinds of disease models or interventions.

## Introduction

The ethics of animal experiments are a matter of a continuing sociopolitical discussion [1]. Frameworks have been implemented in many countries strictly regulating the decision-making process for animal experimentation allowances. However, the application of these regulations struggles with the lack of scientific evidence for the animal’s burden, suffering or distress associated with specific interventions and models as an information basis for harm-benefit analysis [2–5]. This, in particular, applies to animal-based neuroscience research considering that neurological disorders are often characterized by psychiatric comorbidities e.g.[6–8]. Any neurological model or modulation of brain function by genetic, pharmacological, optogenetic stimulation or lesion approaches may influence the affective state of the animal contributing to the burden of an experimental study. Thus, evidence-based severity assessment in neuroscience research requires tailored and sensitive approaches developed to provide comprehensive information about behavioral, biochemical, and/or physiological parameters, which reflect distress or an altered emotional state of the animals. On the other hand, classification of severity needs to meet requirements of practicability and feasibility for the daily management of animal facilities [9].

Thus, approaches for the selection of appropriate and informative evidence-based severity assessment parameters and for the development of composite measure schemes are urgently needed [9]. Focusing on animal models with induced or spontaneous seizures, we provide an example of an approach starting with a large set of candidate parameters assessed in different models and a selection process based on multivariate statistical analyses. The models have been chosen as a practice example based on the obvious limitations in our current understanding of the burden of repeated seizures in laboratory rodents as well as the fact that seizures are a frequent event in different models of various neurological disorders, in breeding populations, in genetically modified animals, and in safety and tolerability studies [10]. Any attempt to translate from human patients needs to consider the different living conditions and the fact that the main burden for patients comes from the unpredictability of seizure occurrence during daily life activities, the psychosocial consequences, the increased risk for physical injury as well as the adverse effects of drugs [11]. While this does not apply to laboratory rodents, other factors require consideration such as invasive electrode and/or transmitter implantation procedures, single housing following implantation, and neurobehavioral comorbidities [8, 10]. The particular need for better knowledge about the impact of respective factors has been emphasized by Lidster et al. (2016) as a basis for evidence-based classification of severity, ethical decisions, animal-welfare based model prioritization, and validation of potential refinement measures.

In the context of a national research consortium aiming to provide better tools for sensitive and robust evidence-based severity assessment [2], we provide a practice example for the process of parameter selection and development of a composite measure scheme designed for precise analysis of the actual distress and suffering of animals. To that end, data from different studies involving three different rat epilepsy models were combined and analyzed to find the top ranking parameters being able to distinguish between control and treated experimental groups. These top-ranking parameters were then used to create a custom composite measure scheme (CMS). This composite measure scheme was subjected to *k*-means clustering which was able to ascribe a severity level to each individual animal. Our findings provide, both, a proof of concept and an applicable composite measure scheme for neurological rat models. The respective online tool for direct application or for CMS generation based on other parameters from different models is available at https://github.com/mytalbot/cms.

## Materials and methods

### Animals

All investigations were in line with the German Animal Welfare Act and the EU directive 2010/63/EU and all reported data is in compliance with the Animal Research: Reporting of In Vivo Experiments (ARRIVE) guidelines. The study was approved by the Government of Upper Bavaria (reference number AZ 55.2-1-54-2531-119-14 and 55.2-1-54-2532-105-16).

The data used in this study was originally published in five individual studies, investigating three different rat epilepsy models [12–16]. A total of seven subprojects were carried out. For all three epilepsy models, severity was assessed using a comprehensive set of behavioral and biochemical parameters. Two additional subprojects were completed for the two post-SE models with additional μPET imaging allowing investigating potential molecular and metabolic imaging biomarker candidates of distress. Moreover, severity was assessed using telemetrically recorded activity and ECG parameters, again to test them as potential measures of distress as well as to test telemetric recordings as putative refinement measures compared to the traditional method of recording using a tethered connection. Table 1 provides an overview of the different original studies and their specific aims and methods. The combined data set comprises findings from 205 female Sprague Dawley rats (Envigo, the Netherlands). All animals were housed individually under controlled conditions (45–65% humidity, 22–24°C, 12-hours, regular light/dark cycle with lights on during day). Food and tap water were available ad libitum. Animals received a fresh Makrolon type III cage with fresh bedding material (Lignocel Select, J. Rettenmaier & Söhne) and nesting material (Enviro-Dri, Claus GmbH, Neuwied Germany) weekly.

**Table 1:** Overview of subprojects and animals used.

Subproject	Model	Monitoring	Techniques	Total n	Publication
1	Electrical Kindling	Not applicable	Behavior, Biochemical	Naïve: 12	[14]
				Sham: 12	
				Treated: 12	
2	Chemical post-SE	Tethered	Behavior, Biochemical	Naïve: 12	[13]
				Sham: 12	
				Treated: 15	
3	Chemical post-SE	Telemetric	Behavior, Biochemical, Telemetry	Sham: 6	[13]
				Treated: 11	
4	Chemical post-SE	Tethered	Behavior, Biochemical, μPET	Naïve: 12	[12]
				Sham: 12	
				Treated: 13	
5	Electrical post-SE	Tethered	Behavior, Biochemical	Naïve: 11	[15]
				Sham: 11	
				Treated: 10	
6	Electrical post-SE	Telemetric	Behavior, Biochemical, Telemetry	Sham: 6	[15]
				Treated: 7	
7	Electrical post-SE	Tethered	Behavior, Biochemical, μPET	Naïve: 12	[16]
				Sham: 8	
				Treated: 9	

### Surgery and epilepsy models

A detailed description of all procedures can be found in respective original publications (Table 1), in short:

#### Electrode and telemetric transmitter implantation

All animals received a bipolar, Teflon-isolated stainless-steel electrode under general anesthesia (i.p. chloral hydrate 360 mg/kg) and local anesthesia (s.c. bupivacaine 0.5%). For perioperative analgesia meloxicam was used (s.c. 1 mg/kg). Electrodes were implanted in either the right Hippocampus (Chemical post-SE model, AP-3.9 mm, L+1.7 mm, DV+4.0/+4.1 mm) or right basolateral amygdala (Electrical kindling & Electrical post-SE model, AP-2.2 mm, L+4.7 mm, DV+8.5 mm).

In two of the five studies (Table 1 studies 3 and 6), the animals received a telemetric transmitter which allowed telemetric recordings of ECG and activity. In addition to the mentioned drugs, animals received locally administered bupivacaine (s.c. Bupivacain 0.5%). Transmitters were implanted subcutaneously at the left abdomen. The negative lead was fixed at the right thorax, the positive lead was fixed 1cm lateral to the sternum at the level of the left rib bow.

#### Electrical kindling model [14]

The kindling procedure was performed according to Russmann et al. [17]. Following a recovery period of three weeks, the afterdischarge threshold (ADT) was determined for each animal. Two days following ADT determination the kindling procedure was initiated. Animals were stimulated daily (5 days a week) with 700 μA (1 msec, monophasic square wave pulses, 50 Hz for 1 s). Suprathreshold stimulation was performed until 10 generalized stage 5 seizures were reached.

#### Chemical post-status epilepticus model [15, 16]

Following a three week recovery period animals were injected with lithium chloride (i.p. 127 mg/kg) 14-16h before pilocarpine treatment. Scopolamine methyl-bromide (i.p. 1 mg/kg) was injected 30 min before pilocarpine injection. Pilocarpine (i.p. 10 mg/kg) was injected every 30 min until the onset of SE (with a maximum of 10 injections). SE was terminated after 90 min by a single injection of phenobarbital (i.p. 25 mg/kg) and multiple injection of diazepam (i.p. 10 mg/kg).

#### Electrical post-status epilepticus model [12, 13]

Status epilepticus was induced according to Walker et al. [18]. Following a recovery period of six weeks, SE was induced by stimulated animals for 25 min in their basolateral amygdala (BLA, intra-train pulse frequency of 50 Hz, 700 μA peak pulse intensity, 100 ms trains of 1 ms alternating positive and negative square-wave-pulses at a frequency of 2 Hz) resulting in self-sustained SE. Four hours following the start of stimulation, SE was terminated by injection of diazepam (i.p. 10 mg/kg) with repeated dosing if necessary.

### Analysis of behavioral and biochemical parameters

**Fig 1:**
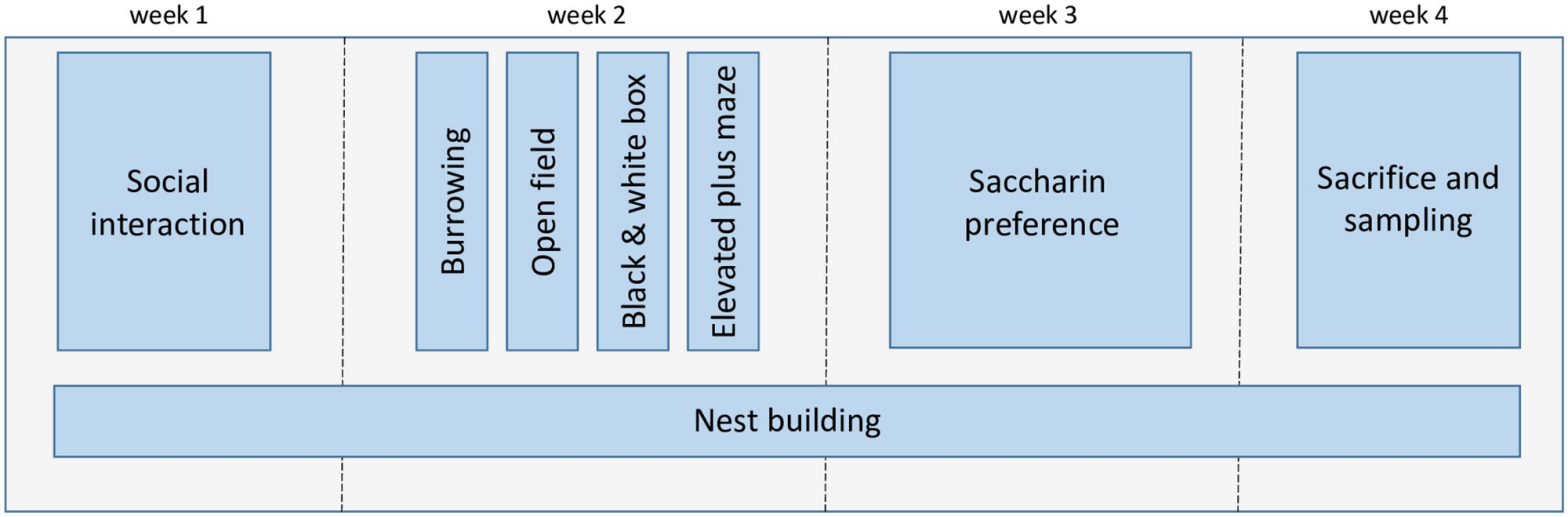
Timeline of behavioral experiments performed.

Throughout the studies, a large set of behavioral, physiological and biochemical parameters were recorded. Depending on the model several parameters were recorded multiple times throughout the different stages of the specific epilepsy model. In this study, we focus on the “final” phase of each epilepsy model, e.g. the chronic phase of the two post-SE models and the phase with generalized seizures of the kindling model. During this phase, the same battery of tests were executed in the same order for all studies. Fig 1 provides an overview of the timeline of the behavioral tests performed. See S1 File for a detailed description of each test.

For all studies included in this study, the same battery of behavioral tests were executed in the same order over a total of four weeks. At the end of each study, animals were sacrificed during which blood and feces samples were taken to investigate the various biochemical parameters.

### Statistics and data analysis

R version 3.6.1 was used for statistics and data analyses [19]. The correlation matrix was visualized using the R package “corrplot” [20], pri ncipal component analysis (PCA) was visualized using the R package “made4” [21], and other graphical representations were prepared using “ggplot2” [22].

All numeric data from individual studies were combined into one data set. The different parameters in this data set were tested for integrity and all parameters with more than 20% missing data were removed. The remaining data were then randomly subdivided into a so-called training (80%) and test set (20%). Each parameter in the training data set was subsequently centered and scaled by subtracting by its mean and dividing by its standard deviations, resulting in a mean of zero and a standard deviation of one. Following a Box-Cox transformation the data was used to perform a PCA. Based on the outcome of the PCA the scaled and centered parameters of interest were summed to create a composite score.

Clusters were calculated using the *k*-means algorithm. Cluster thresholds were determined by 100-fold resampling of the full data into training (80%) and test (20%) data, storing the outer values of each clustering result. These values were used to calculate robust mean cluster thresholds as well as their 95% confidence borders to indicate potential variance instability due to random sampling. Further, at each iteration, information on the ordered eigenvalues of the PCA was used to identify the top-ranking parameters for PC1 and PC2. The PCA analysis provides the (co-)variance information for any number of provided input classes. In order to obtain these relations, we employ a frequency analysis of the most stable features after random subsampling to assess the best set of features for any given data set. This is an unsupervised technique. The ROC analysis for example would require a prior supervised step. Additionally, we show intrinsic class similarity. For this purpose, PCA is not only faster but also much more efficient. Further, we do not use a binary classifier to assess distinct class memberships. Also, depending on the input data the number of classes may change, so that the general problem is no longer binary but multiple. Moreover, we do not have “before intervention” and “after invention” conditions which is a main limitation for using conditional discriminators like logistic regression.

## Results

### Combining and filtering data

The data of seven individual subprojects (results of which are published in five publications, see Table 1) were collected and combined. This combined data set contained 205 animals with a total of 63 different parameters measured in the chronic phase of each epilepsy model. A subset of these parameters was only measured in single projects (e.g. μPET parameters, kindling parameters or parameters recorded telemetrically). For the creation of a composite score, we focussed on parameters measured across all projects. To filter out parameters measured only in individual projects all parameters with more than 20% missing data were removed, resulting in 15 remaining parameters. Of these 15 parameters two were deemed not suitable for a composite severity score: change in body weight, as seizure activity in the models used, is associated with hyperphagia and weight gain, and seizure frequency, as it is a measure of disease phenotype and is only present in the epileptic animals. After all, this resulted in a final selection of 13 numeric parameters including nest building, burrowing, social interaction, saccharin preference, brain derived neurotrophic factor (BDNF) and different parameters investigated in the open field and the black-white box. See S1 File (Table 2 in S1 File) for a detailed list of the parameters.

### Correlation matrix

**Fig 2:**
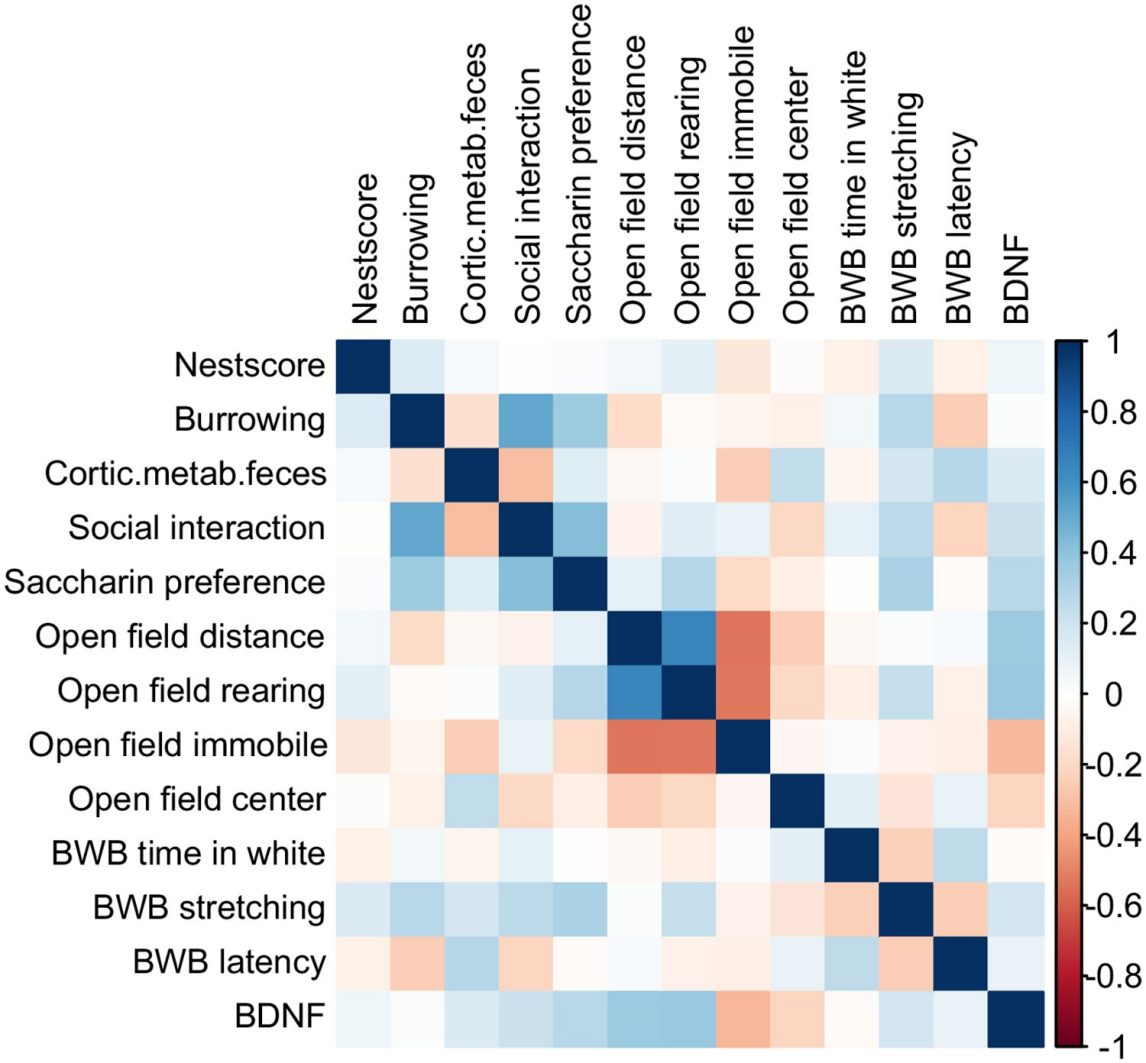
Correlation matrix of selected parameters.

To gain first insight into the selected parameters their distribution was checked and determined to be non-normal. The Spearman correlation coefficients between the parameters were calculated and visualized in a heatmap (Fig 2). While the analysis demonstrated a significant correlation for several parameter combinations (Table 1 in S1 File), only a few parameter combinations exhibited a strong correlation with correlation coefficients either lower than -0.5 or higher than 0.5. Respective parameter combinations comprised burrowing behavior and time spent in social interaction (0.51), as well as different combinations of open field parameters including distance, moved with frequency of rearing postures (0.65) or with time spent in immobility (-0.54) as well as time spent in immobility with frequency of rearing postures (-0.53). These findings indicate that the majority of parameters from different paradigms might rather complement each other to provide more comprehensive information than analysis of a single parameter. However, for practical reasons a selection of parameters is necessary.

Spearman correlation coefficients were calculated for the 13 parameters included in the PCA and following parameter selection. Calculations were performed for the combined data of all animals of all sub-studies. Significant correlations were found between burrowing behavior and time spent in social interaction (0.51), as well as different combinations of open field parameters including distance moved with frequency of rearing postures (0.65) or with time spent in immobility (-0.54) as well as time spent in immobility with frequency of rearing postures (-0.53).

### Principal component analysis

**Fig 3:**
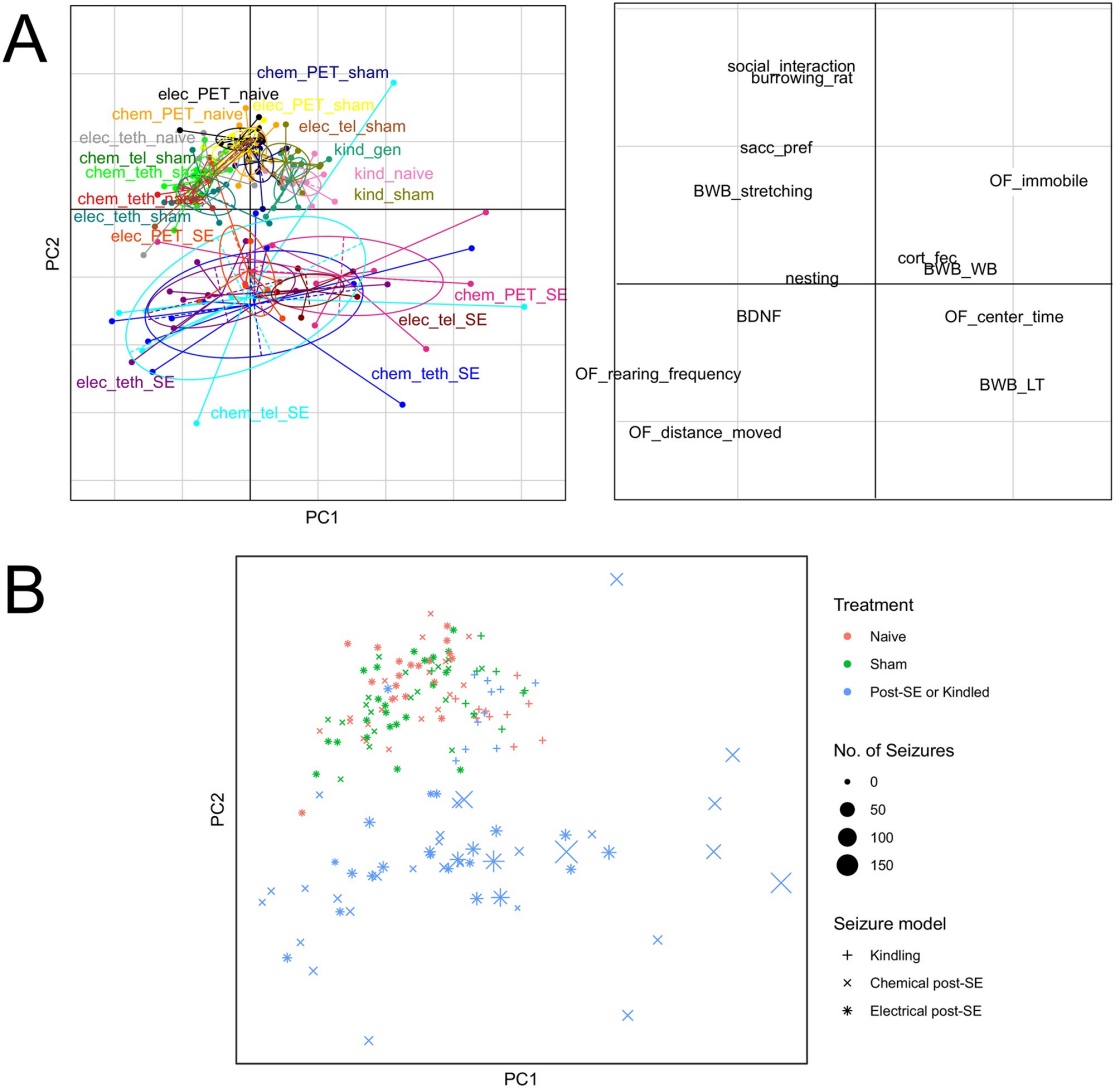
Principal component analysis of selected parameters. A principal component analysis was performed 100 times using a resampled random subset of 80% of the total data. Overall runs PC2 was consistently able to separate the treatment groups on the basis of four parameters: saccharin preference, burrowing behavior, time spent in social interaction and distance moved in the open field. (A) One representative visualization of the principal component analysis. (B) Scatter plot of individual animals and their associated treatment group along PC1 and PC2, the size of the point indicates the number of seizures observed during a two-week monitoring phase.

A PCA was performed 100 times on resampled 80% of the data, referred to as training data. For each run, metrics of the principal components were collected. Fig 3A shows the PCA from one representative run. Over all runs the mean percentage variance on PC1 was 23.62% (SD 0.82%) and on PC2 17.48% (SD 0.51%). In the PCA plots, it can be seen that the separation between the experimental and control group can be found along PC2 (Fig 3A). This separation proved to be consistent across the different repetitions. PC1, on the other hand, does not show a clear relationship with group differences, the variation along PC1 is predominantly caused by open field parameters (i.e. time spent immobile, number of rearing postures, distance moved and time spent in center). One possible relationship between PC1 and individual animals can be observed in Fig 3B, here post-SE animals with a higher seizure frequency during the monitoring phase all have higher PC1 scores, indicating that PC1 can be seen as a measure of disease phenotype in epileptic animals and therefore is not suggested to be used for overall severity assessment. When investigating the loadings of PC2 (Fig 3A) it is apparent that the top five parameters are consistently dominated by the following parameters: saccharin preference, burrowing behavior, time spent in social interaction and two parameters measured in the open field, i.e. distance moved and time spent in immobility. When assessing the top four parameters across the 100 runs, burrowing behavior and social interaction are present in the top four parameters in all 100 runs (i.e. they each make up 25% of the combined parameters). Distance moved in the open field and saccharin preference proved to be among the top four parameters in 99 out of 100 runs (24.75% of the combined parameters), and 60 out of 100 runs (15%), respectively. The remaining parameters, which are among the top four parameters in individual runs all represent a significantly smaller portion: latency to enter the black box in the black and white box (1%), time spent in immobility in the open field (7%), and number of rearing postures (2.25%). The most stable parameters are relatively invariant against resampling, i.e. adding more than four parameters results in an increasingly fragmented set of parameters with no clear favorites beyond the top four mentioned.

Considering the fact that we had to exclude some parameters that were not analyzed in all subprojects, we repeated the analysis with a longer list of parameters that were assessed in sub-projects 1, 2 and 5. When focusing on these subprojects the list of parameters with less than 20% missing data comprised 19 parameters. These 19 parameters included nest building, burrowing, different parameters investigated in the open field, the black-white box and the elevated-plus maze, BDNF, creatinkinase, corticosterone metabolites in feces, saccharin preference and social interaction (Table 2 in S1 File). The PCA-based selection of top parameters again comprised social interaction, burrowing and saccharin preference, which were among the top five parameters. In addition, two parameters from the black-white box were among the top five parameters of this additional analysis. As these were among the 13 parameters analyzed in all subprojects, and were not suggested by the respective PCA-analysis, which was based on more comprehensive data sets, we did not further consider these black-white box parameters. Thus, taken together the additional analysis of a more comprehensive list of parameters in a smaller number of studies did not point to any other parameters of interest.

### Composite score

The choice of parameters used in the composite score was led by the ordered outcome of the PCA with consideration of data from all subprojects. Throughout the 100 runs, PC2 of the PCA (Fig 3A) consistently captured variation which was able to separate the treatment groups. The top parameters being able to do so comprise burrowed weight in the burrowing test, distance moved in the open field, time spent in social interaction, and saccharin preference. In terms of real-life applications, the added benefit of these parameters is that they represent a wide spectrum of behavior while still being non-invasive and relatively easy to apply. This is in part the reason for the number of parameters included in the composite score, as only selecting the top three parameters (e.g. burrowing behavior, open field, and social interaction) would exclude saccharin preference, which is a key measure of anhedonia in rodents [23, 24]. While at the same time the fifth parameter in the top loadings of PC2 is time spent in immobility in the open field which is highly negatively correlated with distance moved in the open field and in addition this fifth parameter did not reach the same level of consistency across the 100 runs of the PCA as the top four parameters.

**Fig 4:**
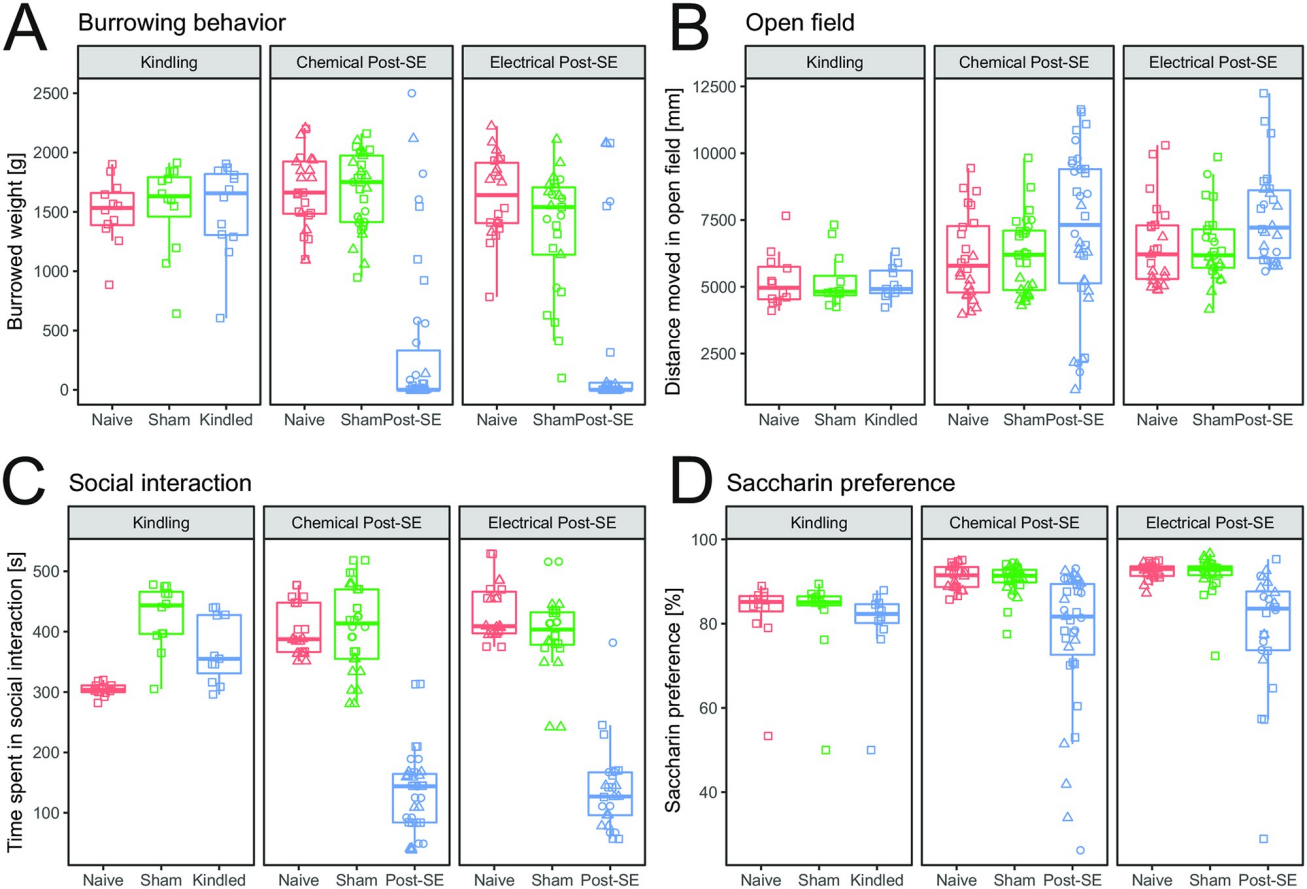
Individual results of selected behavioral parameters. On the basis of the outcome of the principal component analysis, four parameters were used to create a composite score: (A) The amount of gravel burrowed in the burrowing test. (B) The distance moved in the open field. (C) Time spent in active social interaction and (D) the percentage of preference for saccharin solution of normal tap water.

The individual data of the four selected parameters all show an overall treatment effect (burrowing behavior, Fig 4A, (F(2,190) = 91.035, p<0.001), distance moved in open field, Fig 4B, (F(2,189) = 4.608, p = 0.0111), time spent in social interaction, Fig 4C, (F(2,184) = 323.98, p<0.001) and saccharin preference, Fig 4D, (F(2,186) = 28.938, p<0.001)). Two of the three parameters show a clear interaction effect where experimental animals in the kindling model do not differ from their control groups but the two post-SE models do. These parameters include burrowing behavior (F(4,190) = 10.616, p<0.001) and social interaction (F(4,184) = 41.19, p<0.001).

For creating the composite score itself, each of the selected parameters was scaled and centered so that the mean of each parameter is 0 and its standard deviation is 1. Following the transformation, the four parameters were simply summed up to create the composite score. This was possible because each parameter severity has the same directionality, i.e. a low score is assumed to represent higher severity.

### Assessing individual severity levels by k-means clustering

The newly created composite score was subjected to *k*-means clustering. The number of clusters was set to three, which is sufficient to classify animal models into meaningful severity scales. Future studies requiring more elaborate levels of severity can apply the same method with a higher number of clusters.

**Fig 5:**
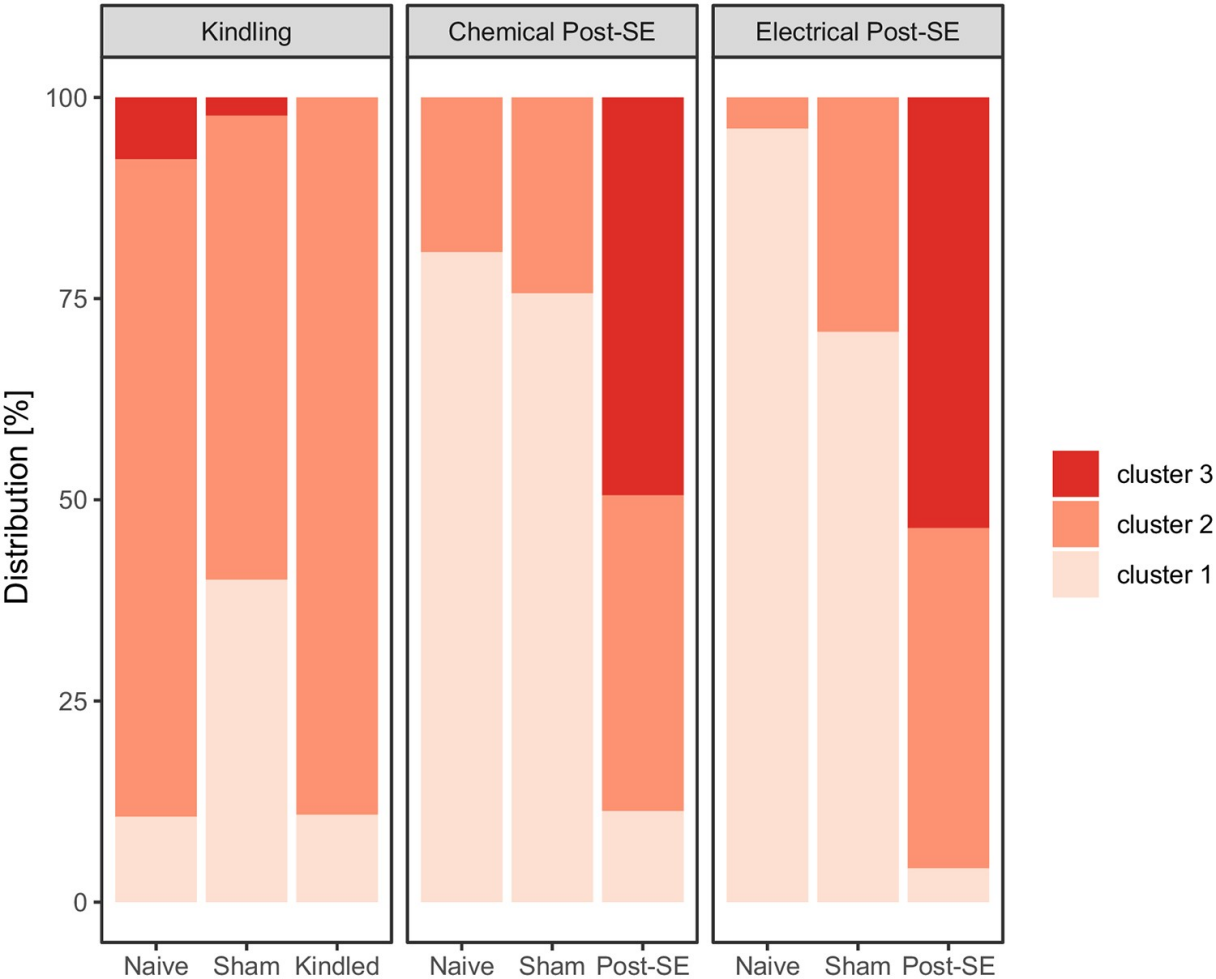
Total distribution of severity clusters per treatment group.

The *k*-means clustering was run 100 times wherein each run each animal was assigned its cluster. Throughout the 100 runs, the overall cluster thresholds were collected. Mean cluster thresholds of the dimensionless scaled data were, from ‘worst’ to ‘best’ or ‘higher’ to ‘lower’ severity, threshold 3 to 2: -2.48 (95% CI: -2.42–-2.54) and threshold 2 to 1: 0.70 (95% CI: 0.67–0.73). Throughout the 100 runs, the distribution of animals and their associated clusters for each treatment and each model were collected. Fig 5 illustrates this distribution to the different clusters. The majority of naive and electrode-implanted sham animals of the studies with post-SE models (chemical and electrical) were classified with cluster 1 (naive: 80.79%, sham: 75.68% and naïve: 96.15%, sham: 70.88%, respectively). Only a small percentage of these control animals were found in cluster 2 (naive: 19.21%, sham: 24.32% and naive: 3.85%, sham: 29.12%, respectively). In the two experimental post-SE groups with chemical and electrical SE induction, around half of the animals were assigned to cluster 3: chemical post-SE 49.43% and electrical post-SE 53.50%. The remaining animals from these groups are mostly classified with cluster 2 (chemical post-SE 39.23% and electrical post-SE 42.28%).

*k*-means clustering was performed on the composite score to assign a severity scale to each individual animal. This was done 100 times using resampled data, during each run the distribution of animals and their associated clusters were collected per treatment group. The bars represent the mean percentage of animals assigned a specific cluster. Overall for the two post-SE models, the two control groups are largely classified with the lowest severity scale while around 50% of the Post-SE animals are associated with the worst severity scale, around 40% of them with the intermediate severity scale and the remaining 10% with the lower severity scale. The Kindling model shows very different results where there is no clear difference between the Kindled animals and their control groups, all groups are largely assigned the intermediate severity scale.

The distribution of the animals from the kindling studies deviates from animals from the post-SE studies (Fig 5). The majority of animals from the control groups and the kindled group are classified with cluster 2 (naive: 81.70% sham: 57.66% and kindling: 89.11%). The remaining animals are mostly found in cluster 1 (naive: 10.65% sham: 40.08% and kindling: 10.89%). Interestingly, a small percentage of control animals from the naive and electrode-implanted group (sham) are classified with cluster 3 (naïve: 7.65% and sham: 2.26%).

The standard deviation of the distribution across all 100 runs for all treatment groups ranges between 5.53% and 11.10%.

For the generation and application of a CMS and the necessary steps “combining and filtering data”, “principal component analysis”, “composite score” and “assessing individual severity levels by k-means clustering” we created a CMS online tool package (https://github.com/mytalbot/cms). This package enables users to create an individual CMS tailored to their parameters, models, and needs or to allocate animals to the given clusters of the reported CMS, which has been developed based on data from epilepsy models.

## Discussion

Evidence-based severity assessment is a presupposition for ethical evaluation of animal test proposals, animal-welfare based model or procedure prioritization, and validation of refinement measures. Considering ongoing socio-political discussions about animal-based research, the development of robust and sensitive severity assessment schemes is also of particular scientific-political relevance [2, 9, 25–27]. Transparent communication about animal-based research should ideally be based on reliable and solid assessment of the burden associated with experimental models and interventions.

Therefore, a national research consortium FOR2591 (https://severity-assessment.de/) has been initiated to develop new strategies for severity assessment [2]. In our laboratory, we have recently completed a comprehensive analysis of behavioral and biochemical parameters in five studies with analysis in three different rat epilepsy models [12–16], which are among the most common models applied in epilepsy research [28]. Multivariate analysis of data sets from these studies resulted in the identification of the most informative parameters, which were integrated into a candidate composite measure scheme (CMS).

According to the ‘EU Directive 2010/63 on the protection of animals used for scientific purposes’ every intervention and procedure in experimental animals needs to be classified as: non-recovery, mild, moderate or severe. An expert working group provided recommendations, which can guide the assignment of a procedure to a particular category with examples of the categorization of different types of procedures (https://ec.europa.eu/environment/chemicals/lab_animals/pdf/report_ewg.pdf). Due to the lack of scientific evidence, these have been largely based on expert opinion. In this context, it has also been pointed out that listing examples might imply the risk that these are applied in a rigid and unquestioning manner without consideration of study- and laboratory-specific influencing factors [27]. Moreover, the classification in categories is not sufficient, when it comes to comparative severity assessment for model comparison or validation of refinement measures. This is, in particular, true as many models and interventions will be categorized as mild or moderate. Thus, a more precise differentiation reflecting more subtle differences in the level of distress is urgently required for comparative severity assessment.

Only recently, efforts have been initiated to develop scientific approaches for sensitive and robust severity assessment strategies [2, 9, 29]. Along this line, the present study provides a practice example on how one can select parameters suitable for severity assessment for a specific category of models. As pointed out above severity assessment in neuroscientific research faces a particular challenge related to the fact that molecular, cellular, and network alterations in the brain may impact the emotional or affective state of the animals [6–8]. Respective alterations are overlooked by common clinical scoring systems, which are mostly focused on body weight, general condition, and activity levels [30, 31]. The conclusion that a more detailed behavioral analysis can bring severity assessment to a different level of sensitivity is underlined by the fact that to our experience a clinical score does not indicate alterations in the majority of animals in the chronic phase of a post-SE model. In apparent contrast, the application of the CMS developed in the present study and of CMS-derived severity levels, indicates interindividual differences in the chronic phase of post-SE models and clearly distinguishes experimental animals with a history of a SE from naïve and electrode-implanted control animals. Interestingly, PCA analysis pointed to a high informative value of behavioral parameters that provide information about changes reflecting the animal’s emotional state. Burrowing has already been intensely discussed as a behavioral pattern, that in mice and rats is not essential for survival under animal facility conditions and that is highly sensitive to the influence of distress and pain with a reduction in the motivation [32–34]. Saccharin preference is a well-established paradigm frequently applied to assess anhedonia in neuropsychiatric research [23, 24]. This parameter has recently been successfully integrated into severity assessment concepts in our previous studies that contributed to the data set used in the present study [12–16]. Activity and social interaction represent parameters that are often integrated into common clinical scores as indicators of animal wellbeing. However, behavioral software-based tracking and analysis allows a more precise and objective assessment, which again contributes to an increased sensitivity. As demonstrated in the present study, the combination of these parameters seems to allow a precise grading of severity levels. Regarding the total number of four parameters integrated into the candidate CMS, we have not only considered the PCA outcome, but also limitations concerning the practicability of the approach.

In the context of parameter selection, it is also interesting to note that these behavioral parameters proved to be superior as compared to biochemical parameters including fecal corticosterone metabolites and serum levels of brain-derived neurotrophic growth factor.

For evaluation of the candidate CMS, we applied k-means algorithm-based cluster analysis. A respective approach has already been used successfully for demarcation of individual severity levels based on analysis of wheel running performance and body weight development in an acute colitis model and a restraint stress model [29]. Cluster analysis with data from all five epilepsy studies revealed that the candidate CMS can guide the categorization in three levels of severity also allowing to assess the distribution of individual animals to different severity levels. In this context, it needs to be considered that these severity levels can be directly applied for comparative severity assessment. While the severity levels can not be directly translated to severity levels in the EU directive 2010/63 without further processing and considerations. These levels allow a direct comparison between models and techniques. Thus, the CMS is a useful tool to compare the severity of models and to compare an intervention or model with and without a possible refinement measure based on a quantitative approach. Regarding rat epilepsy models with induced or spontaneous seizures, our data for instance confirm a higher severity of post-SE models as compared to the kindling paradigm. This finding is in line with previous descriptions of the extent of behavioral alterations in these paradigms [35–38]. Conclusions from this finding should take into account that with once-daily seizure induction the majority of kindled animals have a comparable seizure density than animals in the post-SE models with a mean of 14.6 and 4.6 per week in the chemical and electrical model. Thus, our results provide scientific evidence that disease-associated pathophysiological mechanisms associated with alterations in the affective state rather than the actual seizures contribute to the burden of animal models with repeated seizures. With regard to the technique of chronic electrode implantation, the findings further support our previous conclusion that this seems to have a rather minor impact on the animals’ wellbeing [12–16] when comparing data to those from naïve animals.

Interestingly, animals with spontaneous seizures in the chronic phase of the electrical and chemical post-SE model exhibited a comparable distribution to the severity levels indicating that the distress associated with both models is in the same range. Taking into account that the pharmacoresponsiveness and predictive validity of the kindling paradigm is comparable to that of post-SE models [39], this finding suggests an animal-welfare based prioritization of the kindling paradigm in the context of the assessment of antiseizure drug candidates. A respective recommendation, of course, needs to consider the fact that the scientific question is of particular relevance for the selection of an appropriate model. For instance, post-SE models cannot be replaced by the kindling paradigm for assessment of antiepileptogenic strategies as this requires the induction of an epileptogenic insult followed by a phase of epileptogenesis resulting in manifestation of chronic epilepsy [40, 41]. Moreover, depending on the hypothesis and aim of a study, behavioral alterations characterizing the animal model might be important as target parameters as these can reflect psychiatric comorbidities in patients [8].

Approaches for comparative severity assessment are also required for the assessment of refinement measures. Recommendations for refinement should be based on evidence that the application of a respective measure indeed lowers severity levels. One major refinement concept that has been suggested by experts from NC3R and the epileptology research field is the replacement of tethered seizure monitoring approaches by telemetric recordings [10]. However, as telemetric recordings require more invasive surgery and the chronic transmitter implant might have an impact as well, it is of utmost relevance to comparing these monitoring approaches regarding severity levels. For a respective comparative assessment, the CMS developed in the present study might serve as a valuable tool guiding conclusions.

Surprisingly, severity grading based on CMS-derived severity levels indicated that a relevant percentage of naive and sham animals from the kindling study are sorted into higher severity levels than respective control groups from the studies focused on post-SE models. As this is even more extreme in naive than in electrode-implanted sham animals, this finding may point to the fact that the time in the facility and adjustment to the facility and husbandry procedures can significantly influence behavioral patterns. Based on the time necessary for the model, behavioral analyses were performed 9, 16 and 23 weeks after arrival in the kindling study, the chemical post-SE study, and the electrical post-SE study, respectively. When checking individual parameters, the predominant parameter standing out, which theoretically would indicate a higher severity in naïve controls than in electrode-implanted and kindled animals is the time spent in social interaction. However, as previously discussed, this has been attributed to a high interest of rats towards the implant of their interaction partner in the early weeks following implantation [14]. Nevertheless, the findings for control animals from the kindling study indicate that one should always include appropriate control groups for direct comparison and that bias may occur as naïve animals might react more intensely to exposure to a new environment and handling in the context of behavioral paradigms.

This is also one reason, why future developments should rather focus on home-cage based automatic analysis of behavioral patterns [9].

To test the sampling variation of the clustering process, a 100-fold cross-validation resampling strategy was chosen. To address possible sampling variation the data was divided into two fractions (20% and an 80% ‘training’ set named in accordance with common machine learning terminology). This, however, does not imply a supervised approach but is merely a necessary step to investigate the robustness of cluster stability based on random sampling. The chosen fractions further represent commonly used and accepted standards for cross-validation techniques. The resulting mean borders for the putative severity levels and the corresponding 95% confidence intervals indicate high cluster stability and will further allow the identification of samples with ambiguous cluster classification. While this method provides first evidence for a good reproducibility and validity of the approach, and the candidate CMS, it will be of utmost relevance to further assess the robustness and the generalizability of this CMS by application to other epilepsy models, studies in animals of different age and sex as well as studies completed in other laboratories. Recently we recommended a classification as ‘severe’ for post-SE models [13, 15]. This recommendation has been based on the transient higher severity during the early days following SE. In the chronic phase with spontaneous recurrent seizures, the severity would be rather considered ‘moderate’ according to the EU Directive. Thus, it will be of relevance that one also assesses the suitability of the score to grade severity at higher levels for instance by assessment during the early post-SE phase. Moreover, the generalizability of the parameter selection procedure should be further evaluated by application to different model categories. In addition, the robustness of the score needs to be assessed in particular considering variables such as the order of the tests or the time span during which the tests are applied.

## Conclusion

In conclusion, we have demonstrated that multivariate data analysis, i.e. principal component analysis, can guide the selection of parameters valuable for severity assessment. The findings pointed to a particular value of behavioral parameters and resulted in the design of a CMS based on assessment of burrowing behavior, open field locomotion, social interaction, and saccharin preference. Cluster analysis confirmed the validity of this candidate CMS allowing categorization in three levels of severity. Assessment of the distribution of individual animals to these different severity levels provided information about interindividual variability of severity for the models and allowed a direct comparison of the severity of the models. The CMS reported here can be directly applied for comparison of other rat models with seizure activity or for assessment of novel refinement approaches in the respective research field. However, the robustness and generalizability needs to be further assessed in future studies. More importantly, our concept of parameter selection can serve as a practice example providing the basis for comparable approaches applicable to the development and validation of CMS for all kinds of disease models or interventions.

## Supporting information

S1 File (DOC)
