# Supplementary material for: Design of composite measure schemes for comparative severity assessment in animal-based neuroscience research: A case study focussed on rat epilepsy models
Source: PLoS One. 2020 May 15;15(5):e0230141. doi: 10.1371/journal.pone.0230141 (PMC7228039; doi:10.1371/journal.pone.0230141)
Supplement: S1 File — (DOC) [file pone.0230141.s001.doc]

**Nest building and latency to nest building**

Nest building behavior was tested as described by Van Loo and Baumans (1) and Jirkof, Fleischmann (2). Before the experiment, the optimal time point for evaluation of peak nest complexity was identified by daily nest scoring during one baseline week. The complexity of the nest and shape of the nesting material (0 = not touched/ destroyed, 1 = flat, 2 = slightly curved, 3 = deep) was scored daily between 7 and 9 a.m. Nest complexity scores peaked at day five following cage changing. Therefore, nest scoring was subsequently carried out once per week at the fifth day following cage changing. During the first following surgery and following SE, scores were determined on a daily basis. In addition, we assessed the level of soiling (1 = clean, 2 = slightly soiled, 3 = substantially soiled) once a week before the animals received fresh embedding material according to the protocol by Van Loo and Baumans (1).

**Social Interaction Test**

The test was performed according to a previously described protocol [3, 4]. Rats were housed individually at least two weeks before testing without previous contact to maximize the time spent in social interaction. On two consecutive days, all animals underwent a habituation procedure during which animals were transferred to the dimly lit (20 lux) behavioral room and placed singly into test cages (Makrolon Type IV) for ten minutes. At the test day, animals were placed in the test arena with a weight-matched partner for ten minutes. A combined score was obtained for each pair. The time spent in active social interaction (sniffing, grooming, play, following, walking on each other) was recorded in seconds.

**Burrowing**

The burrowing test was carried out as previously described [5, 6]. Plastic burrowing tubes (32 cm long x 10 cm Ø, elevated on one site by 6 cm) were filled with 2.5 kg gravel (quartz-light, grain size 2 - 4 mm, ORBIT GmbH, Germany) and placed in a Makrolon Type IV cage. Every rat was tested separately. All rats received a training prior to the assessment of baseline (before surgery) and experimental data. On day 1 of the training phase, rats received an empty burrowing tube for 60 minutes; on day 2 - 4, rats received a gravel-filled tube for 60 minutes after a habituation time of 30 minutes in the empty cage. Burrowing behavior was tested on day 5 (baseline), one week after surgery as well as one, four and eleven weeks following status epilepticus. As soon as the burrowing tube was placed in the cage the latency time to start burrowing was measured. The remaining gravel in the tubes was weighed and the difference to the initial value was calculated to determine the displaced gravel. Two animals that burrowed less than 500 g at the day of the baseline evaluation were excluded from further analysis.

The open field test, black-white box, and elevated plus maze were carried out as previously described [7], in short:

**Open field test**

The open field test was used to determine locomotor activity of the animals. The animals were placed in a round open field (Ø= 85 cm, 10-20 lux) facing the wall from a distance of 10 cm and were then recorded for 10 minutes. Activity parameters (distance moved, immobility time, rearing frequency, and time in each zone (wall and center)) were assessed using a tracking software (EthoVision, Version 8.5, Noldus, Wageningen, Netherlands).

**Black-white box**

A plastic box with a black (39 x 20 x 39 cm, with a top) and a white (39 x 39 x 39 cm, 50 lux) compartment, connected through a poorly illuminated tunnel was used. The animals were placed in the white compartment facing the tunnel. The behavior (e.g. latency to enter the black box, stretching postures, time spent in the black and the white box) was recorded for five minutes.

**Elevated plus maze**

The apparatus for the elevated plus maze [8] consisted of a central platform (14 x 14 cm), two open arms (50 x 14 cm, 20 lux), and two closed arms (15 x 14 x 29 cm, 10 lux). The maze was elevated 82 cm above the ground. The animals were placed in the center of the maze facing the same closed arm. We recorded the behavior for five minutes to score anxiety-associated behavior.

**Saccharin preference test**

The saccharin preference test was performed in accordance with a protocol described by Klein, Bankstahl (9). Two water bottles were attached to the cage. 500 g of the solutions were provided in each water bottle (total volume 700 ml, 70 x 70 mm square, Ehret Labor- und Pharmatechnik, Germany) with a watering nibble with a diameter of 1 mm. Initially, we evaluated water intake from both bottles over 24 hours in order to assess a putative side preference. Next, one of the two bottles provided was filled with 0.1% saccharin solution (Aldrich Saccharin ≥ 98%, Sigma-Aldrich Chemie GmbH, Germany) and the intake was determined for another 24 hours. At the third day both bottles were filled again with regular water and at the fourth day the saccharin-containing solution was provided in the bottle on the other side.

**Analysis of hair corticosterone**

Hair from all animals was shaved in the neck (approx. 4 x 4 cm) before the start of the experiments to exclude an impact of previous events on the data. Immediately before surgery, the regrown hair was shaved and these hair samples were used to determine a baseline value for the corticosterone levels. Afterwards the hair was allowed to grow again during the experiment with status epilepticus induction and subsequent behavioral assessment. Hair samples were collected at the end of the project. All hair samples were stored at room temperature excluded from light. The samples were analyzed as described before by Gao, Stalder (10) with liquid chromatography tandem mass spectrometry (LC-MS/MS). Corticosterone levels are given in pg/mg.

**Analysis of fecal corticosterone metabolites**

Feces samples were collected before electrode-implantation to determine a baseline value. Further samples were collected two days as well as one, four and 13^th^ weeks following status epilepticus. Animals were placed in a fresh cage in the morning (7 to 9 a.m.) to collect fresh feces during the subsequent three hours. Feces were stored frozen at -20°C. 80% methanol was used for extraction. The analysis of the fecal corticosterone metabolites was carried out by an enzyme immunoassay (EIA) as described before by Lepschy, Touma (11). Concentrations of fecal corticosterone metabolites are provided in ng/g.

**Serum analysis**

Blood was only sampled at the end of the experiment to minimize an impact of the invasive procedure on severity assessment in this study. Blood was collected by retrobulbar puncture under isoflurane anesthesia in the morning (9 to 10:30 a.m.) one day before perfusion. Animals were allowed to habituate to the room for at least 30 minutes before blood sampling. Blood was collected in Eppendorf vials and left for coagulation for 45 to 60 minutes. Afterwards, the blood was centrifuged at 1500 x g for 10 minutes and stored at -80°C.

Serum corticosterone levels were determined by an enzyme-linked immunosorbent assay (ELISA) (Corticosterone rat/mouse ELISA, DEV9922, Demeditec). The absorbance was measured at 450 nm with an ELISA reader (Gen 5 microplate reader, Biotek; Gen 5 Imager Software, Biotek, Germany) with a four-parameter logistic curve program for calculation of the corticosterone concentration in the samples. Corticosterone levels are presented in ng/ml. Analysis of corticosterone failed in the tethered subproject for one animal of the sham group and in one animal of the naive group for unknown reasons.

BDNF concentrations were measured in serum samples (1:20 diluted with sample buffer) using a highly sensitive and specific fluorometric two-site ELISA according to the manufacturer’s instructions (Promega Inc, Germany). The exact procedure has been described in detail previously by Hellweg, von Arnim (12) and Deuschle, Gilles (13). BDNF levels are provided as pg/ml.

**Table 1. P values for correlation matrix in Figure 2.**

**Table 2. Overview of parameter analyzed in the different models and subprojects.**

|  | 13 parameters for parameter selection |  |  | Electrical kindling [14] | Chemical post-SE | | | Electrical post-SE | | |
| --- | --- | --- | --- | --- | --- | --- | --- | --- | --- | --- |
|  |  |  |  |  | Teth.[15] | Teth. + PET[16] | Tel.[15] | Teth.[17] | Teth. + PET[18] | Tel.[17] |
| Behavioral parameters | x | Nest building |  | √ | √ | √ | √ | √ | √ | √ |
|  | x | Burrowing |  | √ | √ | √ | √ | √ | √ | √ |
|  |  | Level of soiling |  | √ | √ |  | √ | √ |  | √ |
|  | x | Open field | Distance  moved | √ | √ | √ | √ | √ | √ | √ |
|  | x |  | Rearing | √ | √ | √ | √ | √ | √ | √ |
|  | x |  | Immobile | √ | √ | √ | √ | √ | √ | √ |
|  | x |  | Center time | √ | √ | √ | √ | √ | √ | √ |
|  | x | Social interaction |  | √ | √ | √ | √ | √ | √ | √ |
|  | x | Saccharin preference |  | √ | √ | √ | √ | √ | √ | √ |
|  | x | Black-white box | Time in white box | √ | √ | √ | √ | √ | √ | √ |
|  | x |  | Stretching | √ | √ | √ | √ | √ | √ | √ |
|  | x |  | Latency | √ | √ | √ | √ | √ | √ | √ |
|  |  | Elevated-plus maze | Closed arms | √ | √ |  | √ | √ | √ | √ |
|  |  |  | Open arms | √ | √ |  | √ | √ | √ | √ |
|  |  |  | Open arms (outer part) | √ | √ |  | √ | √ | √ | √ |
|  |  |  | Stretching | √ | √ |  | √ | √ | √ | √ |
|  |  |  | Head dip | √ | √ |  | √ | √ | √ | √ |
| Biochemical parameters | x | BDNF |  | √ | √ | √ | √ | √ | √ | √ |
|  | x | Corticosterone | metabolites feces | √ | √ | √ | √ | √ |  | √ |
|  |  |  | Hair | √ | √ | √ |  | √ |  |  |
|  |  |  | Serum | √ | √ | √ | √ | √ | √ | √ |
|  |  | Creatinkinase | Serum | √ | √ | √ |  | √ | √ |  |
|  |  | Oxytocin | Serum | √ |  | √ |  |  | √ |  |
|  |  | Adrenal gland weight |  | √ | √ | √ | √ | √ | √ | √ |
| µPET parameters |  | MPPF (Serotonin-1A receptor ligand) | Hippo-campus (left + right) |  |  | √ |  |  | √ |  |
|  |  |  | Medial prefrontal cortex |  |  | √ |  |  | √ |  |
|  |  |  | Septum |  |  | √ |  |  | √ |  |
|  |  | FDG | Hippo-campus (left + right) |  |  | √ |  |  | √ |  |
|  |  |  | Medial prefrontal cortex |  |  | √ |  |  | √ |  |
|  |  |  | Septum |  |  | √ |  |  | √ |  |
|  |  |  | Amygdala |  |  | √ |  |  | √ |  |
|  |  |  | Striatum |  |  | √ |  |  | √ |  |
|  |  |  | Hypo-thalamus |  |  | √ |  |  | √ |  |
|  |  |  | Thalamus |  |  | √ |  |  | √ |  |
| Telemetric parameters |  | Heart rate |  |  |  |  | √ |  |  | √ |
|  |  | Activity |  |  |  |  | √ |  |  | √ |
|  |  | Heart rate variablity | NN-I |  |  |  | √ |  |  | √ |
|  |  |  | NNx |  |  |  | √ |  |  | √ |
|  |  |  | NNx |  |  |  | √ |  |  | √ |
|  |  |  | RMSSD |  |  |  | √ |  |  | √ |
|  |  |  | SDNN |  |  |  | √ |  |  | √ |
| Seizure-related parameters |  | Spontaneous seizures | Seizures_n |  | √ | √ | √ | √ | √ | √ |
|  |  |  | Seizure duration |  | √ | √ | √ | √ | √ | √ |
|  |  | Induced seizures | Initial after- discharge threshold | √ |  |  |  |  |  |  |
|  |  |  | Initial seizure duration | √ |  |  |  |  |  |  |
|  |  |  | Total seizure duration | √ |  |  |  |  |  |  |
|  |  |  | Mean seizure duration | √ |  |  |  |  |  |  |

√ = assessed in the model/subproject
Teth. = tethered EEG recording
Tel. = telemetric EEG recording

**References**

1. Van Loo PL, Baumans V. The importance of learning young: the use of nesting material in laboratory rats. Lab Anim. 2004;38(1):17-24. Epub 2004/02/26. doi: 10.1258/00236770460734353. PubMed PMID: 14979984.

2. Jirkof P, Fleischmann T, Cesarovic N, Rettich A, Vogel J, Arras M. Assessment of postsurgical distress and pain in laboratory mice by nest complexity scoring. Lab Anim. 2013;47(3):153-61. Epub 2013/04/09. doi: 10.1177/0023677213475603. PubMed PMID: 23563122.

3. Hölter SM, Einicke J, Sperling B, Zimprich A, Garrett L, Fuchs H, et al. Tests for Anxiety-Related Behavior in Mice. Current protocols in mouse biology. 2015;5(4):291-309. Epub 2015/12/03. doi: 10.1002/9780470942390.mo150010. PubMed PMID: 26629773.

4. File SE, Lippa AS, Beer B, Lippa MT. Animal tests of anxiety. Curr Protoc Neurosci. 2004;Chapter 8:Unit 8 3. Epub 2008/04/23. doi: 10.1002/0471142301.ns0803s26. PubMed PMID: 18428606.

5. Rutten K, Robens A, Read SJ, Christoph T. Pharmacological validation of a refined burrowing paradigm for prediction of analgesic efficacy in a rat model of sub-chronic knee joint inflammation. European journal of pain (London, England). 2014;18(2):213-22. Epub 2013/07/16. doi: 10.1002/j.1532-2149.2013.00359.x. PubMed PMID: 23852581.

6. Rutten K, Schiene K, Robens A, Leipelt A, Pasqualon T, Read SJ, et al. Burrowing as a non-reflex behavioural readout for analgesic action in a rat model of sub-chronic knee joint inflammation. European journal of pain (London, England). 2014;18(2):204-12. Epub 2013/07/16. doi: 10.1002/j.1532-2149.2013.00358.x. PubMed PMID: 23853119.

7. Pekcec A, Muhlenhoff M, Gerardy-Schahn R, Potschka H. Impact of the PSA-NCAM system on pathophysiology in a chronic rodent model of temporal lobe epilepsy. Neurobiol Dis. 2007;27(1):54-66. Epub 2007/05/22. doi: 10.1016/j.nbd.2007.04.002. PubMed PMID: 17513116.

8. File SE. The interplay of learning and anxiety in the elevated plus-maze. Behav Brain Res. 1993;58(1-2):199-202. Epub 1993/12/20. doi: 10.1016/0166-4328(93)90103-w. PubMed PMID: 8136046.

9. Klein S, Bankstahl JP, Loscher W, Bankstahl M. Sucrose consumption test reveals pharmacoresistant depression-associated behavior in two mouse models of temporal lobe epilepsy. Experimental neurology. 2015;263:263-71. Epub 2014/09/16. doi: 10.1016/j.expneurol.2014.09.004. PubMed PMID: 25220610.

10. Gao W, Stalder T, Foley P, Rauh M, Deng H, Kirschbaum C. Quantitative analysis of steroid hormones in human hair using a column-switching LC-APCI-MS/MS assay. Journal of chromatography B, Analytical technologies in the biomedical and life sciences. 2013;928:1-8. Epub 2013/04/16. doi: 10.1016/j.jchromb.2013.03.008. PubMed PMID: 23584040.

11. Lepschy M, Touma C, Palme R. Faecal glucocorticoid metabolites: how to express yourself - comparison of absolute amounts versus concentrations in samples from a study in laboratory rats. Lab Anim. 2010;44(3):192-8. Epub 2010/01/15. doi: 10.1258/la.2009.009082. PubMed PMID: 20071410.

12. Hellweg R, von Arnim CA, Buchner M, Huber R, Riepe MW. Neuroprotection and neuronal dysfunction upon repetitive inhibition of oxidative phosphorylation. Experimental neurology. 2003;183(2):346-54. Epub 2003/10/14. doi: 10.1016/s0014-4886(03)00127-4. PubMed PMID: 14552876.

13. Deuschle M, Gilles M, Scharnholz B, Lederbogen F, Lang UE, Hellweg R. Changes of serum concentrations of brain-derived neurotrophic factor (BDNF) during treatment with venlafaxine and mirtazapine: role of medication and response to treatment. Pharmacopsychiatry. 2013;46(2):54-8. doi: 10.1055/s-0032-1321908. PubMed PMID: 22961097.

14. Möller C, Wolf F, van Dijk RM, Di Liberto V, Russmann V, Keck M, et al. Toward evidence-based severity assessment in rat models with repeated seizures: I. Electrical kindling. Epilepsia. 2018;59(4):765-77. Epub 2018/02/27. doi: 10.1111/epi.14028. PubMed PMID: 29479675.

15. Koska I, van Dijk RM, Seiffert I, Di Liberto V, Möller C, Palme R, et al. Toward evidence-based severity assessment in rat models with repeated seizures: II. Chemical post-status epilepticus model. Epilepsia. 2019. Epub 2019/09/01. doi: 10.1111/epi.16330. PubMed PMID: 31471910.

16. Di Liberto V, van Dijk RM, Brendel M, Waldron AM, Möller C, Koska I, et al. Imaging correlates of behavioral impairments: An experimental PET study in the rat pilocarpine epilepsy model. Neurobiol Dis. 2018;118:9-21. Epub 2018/06/23. doi: 10.1016/j.nbd.2018.06.010. PubMed PMID: 29933054.

17. Seiffert I, van Dijk RM, Koska I, Di Liberto V, Moller C, Palme R, et al. Toward evidence-based severity assessment in rat models with repeated seizures: III. Electrical post-status epilepticus model. Epilepsia. 2019;60(8):1539-51. Epub 2019/06/28. doi: 10.1111/epi.16095. PubMed PMID: 31247135.

18. van Dijk RM, Di Liberto V, Brendel M, Waldron AM, Möller C, Gildehaus FJ, et al. Imaging biomarkers of behavioral impairments: A pilot micro-positron emission tomographic study in a rat electrical post-status epilepticus model. Epilepsia. 2018;59(12):2194-205. Epub 2018/10/30. doi: 10.1111/epi.14586. PubMed PMID: 30370531.
